# Supplementary material for: Environmental DNA reveals seasonal shifts and potential interactions in a marine community
Source: Nat Commun. 2020 Jan 14;11:254. doi: 10.1038/s41467-019-14105-1 (PMC6959347; doi:10.1038/s41467-019-14105-1)
Supplement: Supplementary file 1 — Supplementary Information [file 41467_2019_14105_MOESM1_ESM.pdf]

Supplementary Information

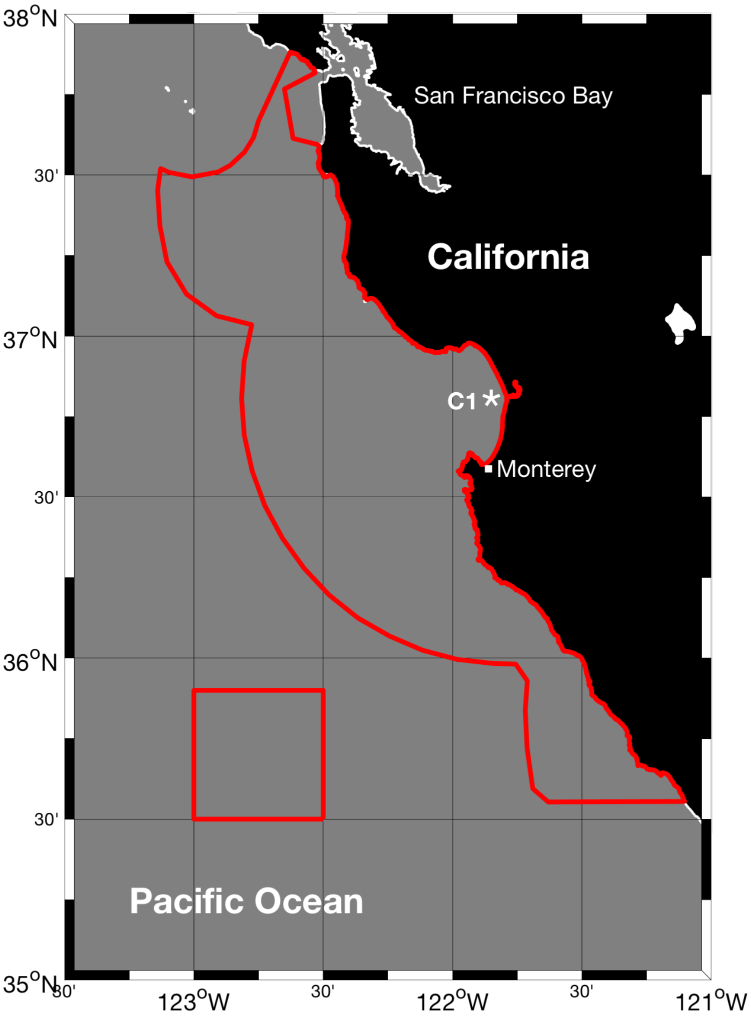

Figure 1. Map of Monterey Bay, California. The red lines represent the boundaries of the Monterey Bay National Marine Sanctuary (MBNMS). The red square outlines the Davidson Seamount boundary, an extension of the MBNMS. The sampling station C1 is marked with an asterisk. The map was produced using the m_map package for MATLAB: Pawlowicz, R., 2019. "M_Map: A mapping package for MATLAB", version 1.4k, [Computer software], available online at www.eoas.ubc.ca/~rich/map.html.

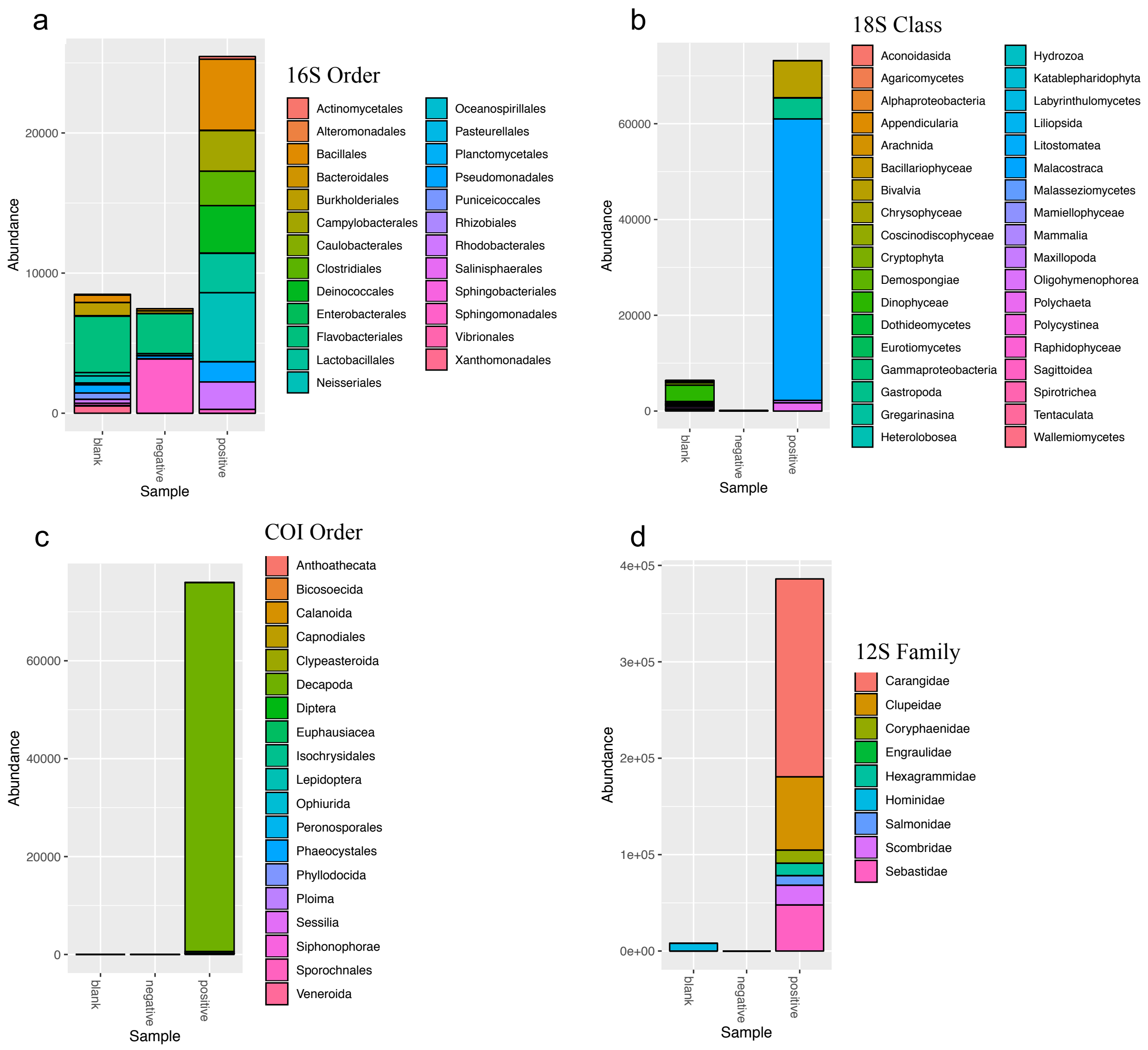

Figure 2. Blanks, negative, and positive controls. Sequence counts of taxa detected in the blanks (DNA extraction and PCR blanks), negatives (sterile water) and positives (artificial communities) for 16S (a), 18S (b), COI (c), and 12S (d). The taxa from each genetic marker were agglomerated at different levels of taxonomy (family-class) for the illustration of this figure, see figure legend.

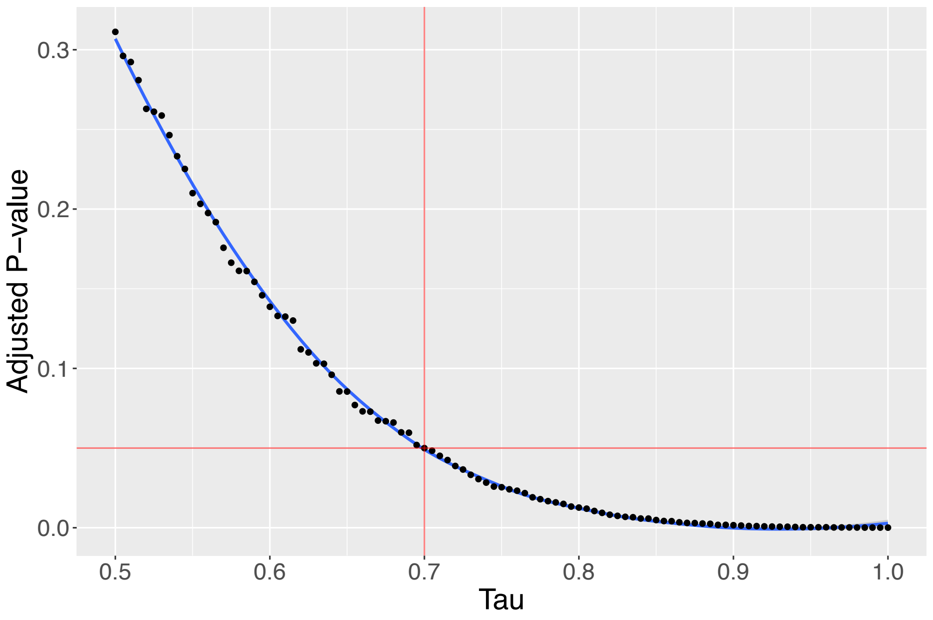

Figure 3. Results from Kendall's tau calculations on taxa correlations. Adjusted P-value against Kendall's tau with the red line representing the cut-off limit of taxa included in the analyses of this manuscript. All taxa below a Kendall's tau of 0.7 were removed from the dataset as these represent non-significant correlations. Significant p-value is set at 0.05.

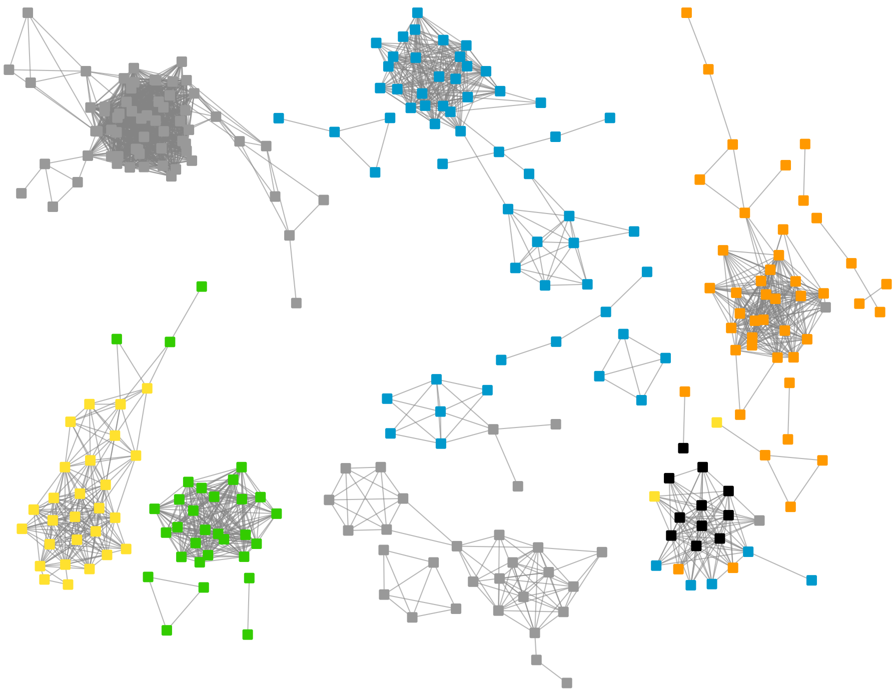

Figure 4. Whole network visualization based on Fig. 1a. The whole network is visualized with nodes (taxa) and edges (correlations) representing the connections between the individual taxa. To best visualize the network only connections with an r > 0.2 are shown.

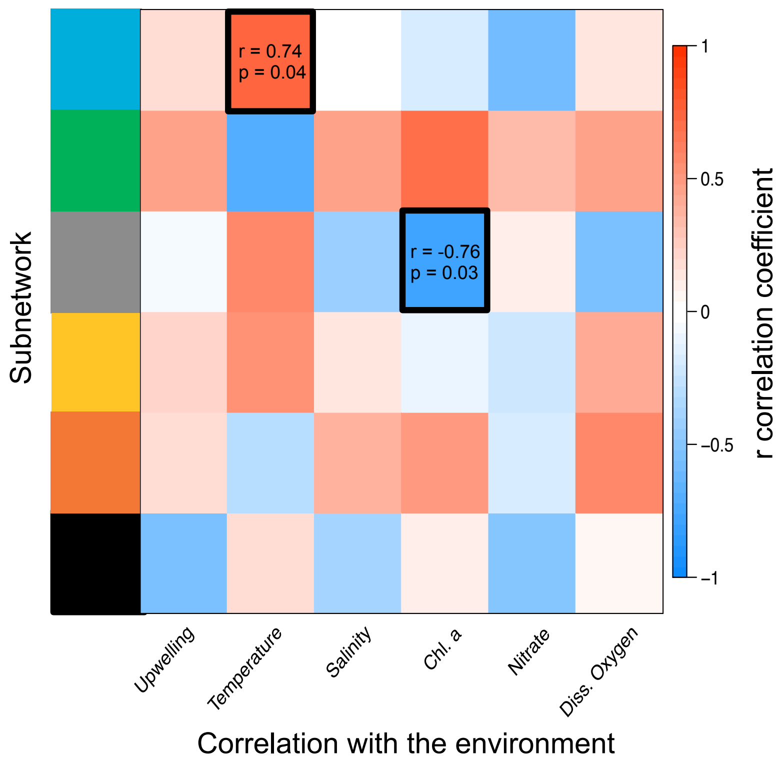

Figure 5. Subnetwork correlation to the environment. All subnetworks, and thus all taxa, were correlated to all the measured environmental parameters in this study. The blue (with highest richness in the winter) and grey (with highest richness in the autumn) were the only subnetworks that were significantly correlated to temperature and chlorophyll *a,* respectively.

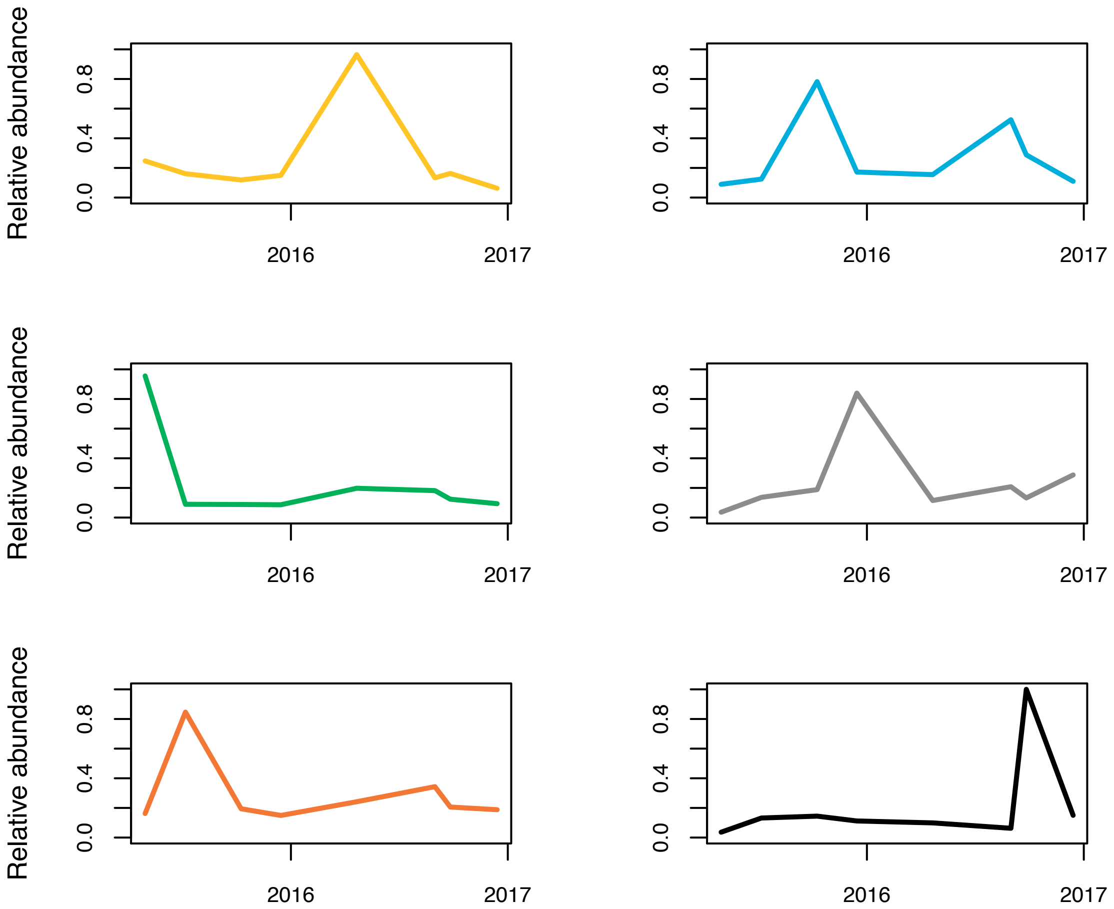

Figure 6. Average amplicon proportional abundance trend of taxa within each subnetwork. The mean amplicon abundance amplicon-index was calculated for each subnetwork by time point. The colours represent subnetwork as divided by the cluster analysis in the main text Figure 1a.

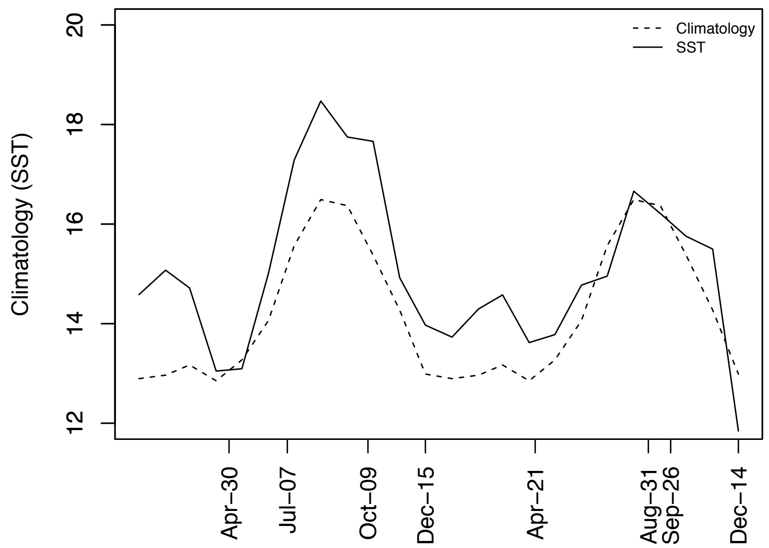

Figure 7. Climatology and sea surface temperature during sampling period. The dashed line represents the climatology (average sea surface temperature) for Monterey Bay, California, and the solid line represents the measured sea surface temperature for our sampling period at station C1 (Supplementary Information Fig. 1).

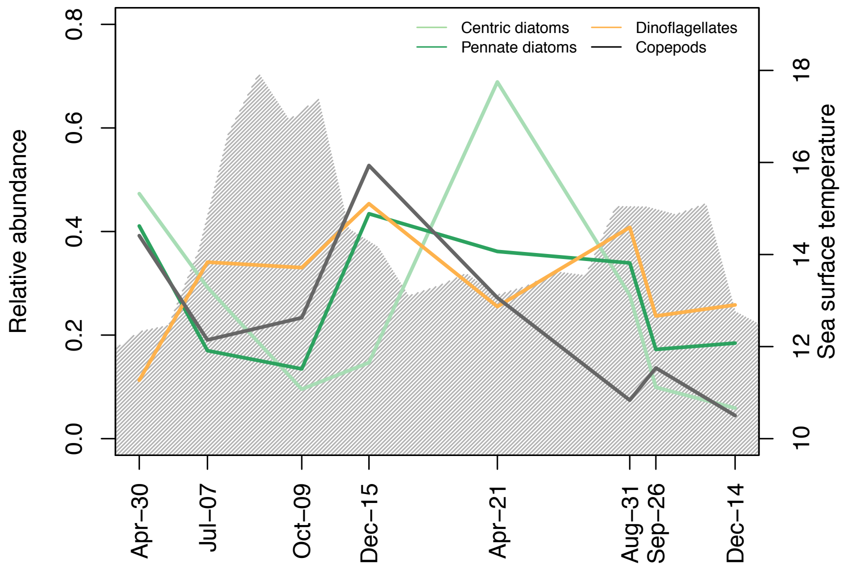

Figure 8. Average trends in phytoplankton and copepods. Centric diatoms, pennate diatoms, dinoflagellates and copepods with time through our sampling season. The average sea surface temperature is shaded in the background (z-axis).

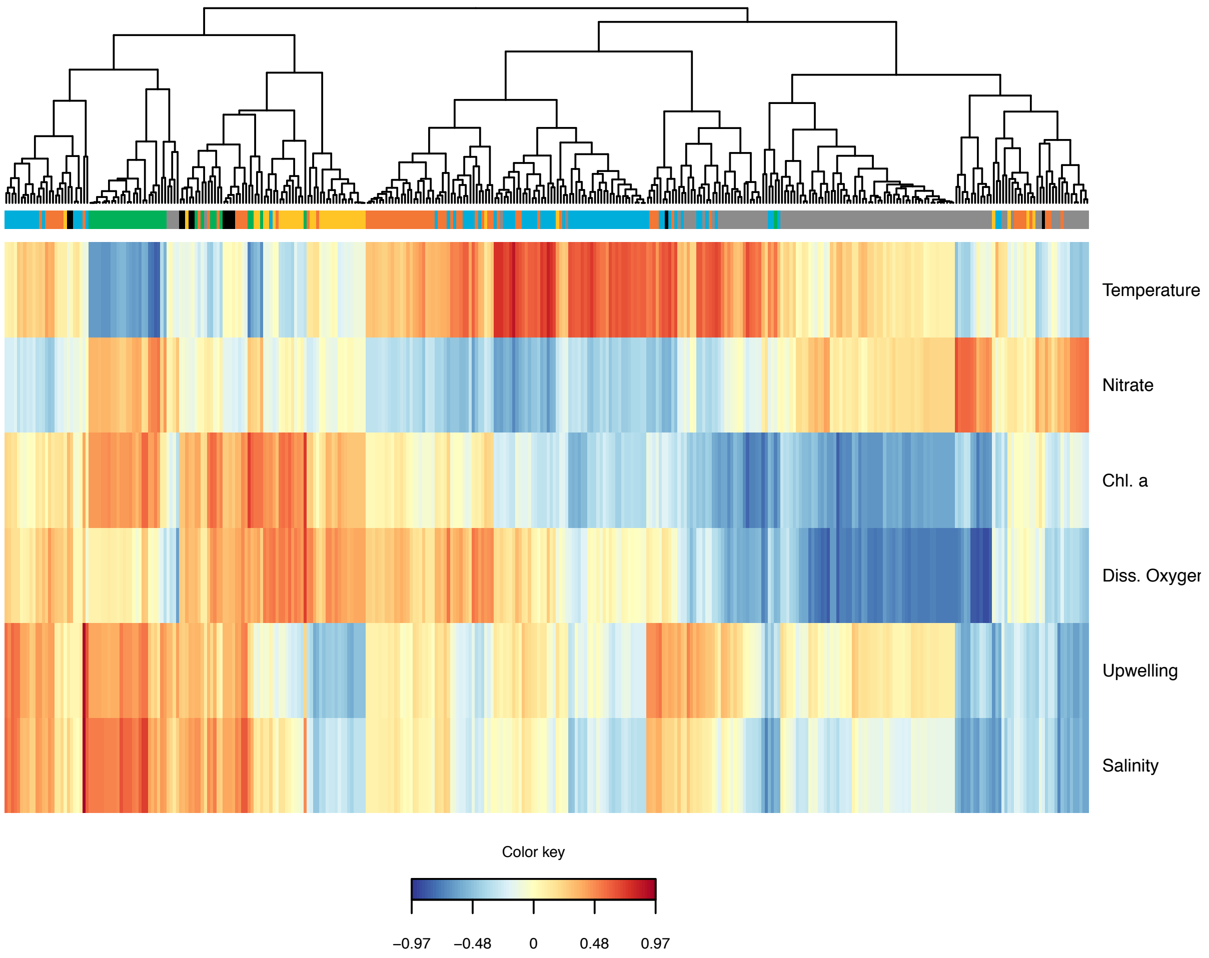

Figure 9. Partial least square analysis between taxa and environmental variables. Partial least square analysis plot showing the correlation of all taxa (columns), clustered by Kendall's tau correlation coefficient, in the yellow, green, black, and orange subnetworks (leaf colour) with environmental variables (rows). The heatmap is coloured by the value of the correlation coefficient, see colour key.

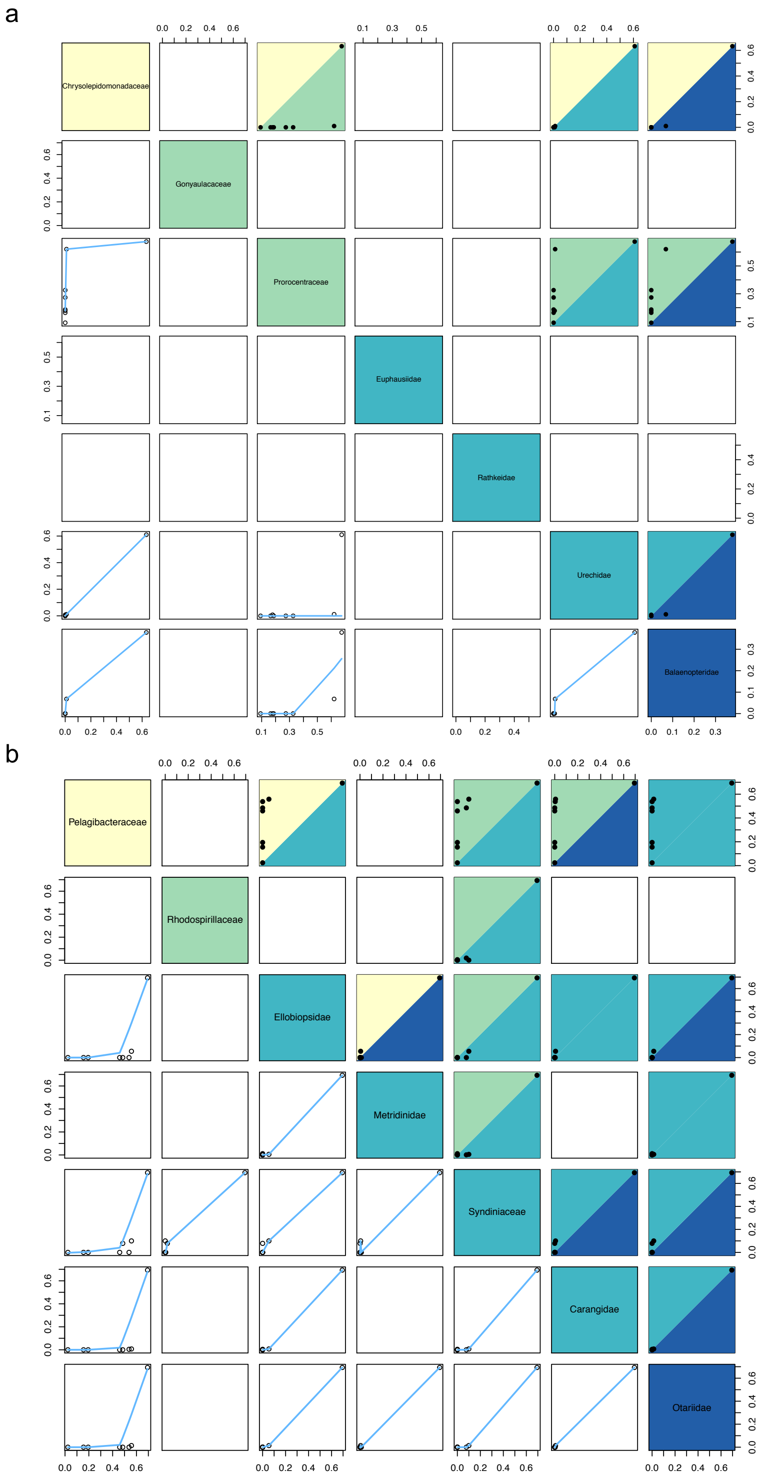

Figure 10. Kendall's tau correlations of taxa coloured by trophic level. Figure 10a shows correlations of all taxa mentioned from the blue network (taxa names are in the diagonal). Figure 10b shows correlations of all taxa mentioned for the grey network. The colours indicate trophic level accordingly: yellow: trophic level 0 (saprotrophs), green: trophic level 1 (primary producers), teal: trophic level 2 (primary consumers), light blue: trophic level 3 (secondary consumers), dark blue: trophic level 4 (tertiary consumers). Only significant correlations ($p<0.05$) are plotted.

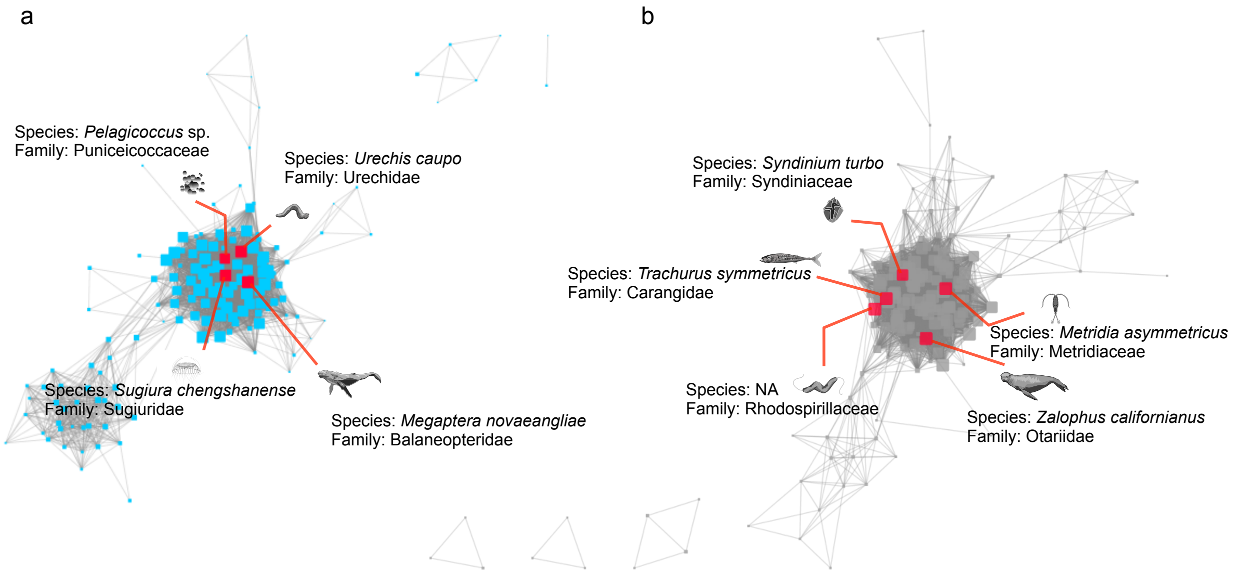

Figure 11. WGCNA network visualization based on taxonomic assignment at the species level. This network is visualized with nodes (species) and edges (correlations) representing the connections between the individual taxa. To best visualize the network only connections with an $r > 0.2$ are shown. All taxa represented in Fig. 2a are presented in the blue network, except Euphausiidae, due to the fact that species-level analysis grouped them in a different subnetwork. The same taxa represented in Fig. 2 are presented here at the species level. Species names are written adjacent to the taxa and the family, corresponding to the annotation in Fig. 2, is written below.

Table 1: Overview of bioinformatics parameters used for each genetic locus.

Step	Program	Parameter	16S rRNA	18S rRNA	COI	12S rRNA
Paired-end merging	PEAR	Min. overlap size	20	50	150	123
		Max. assembly length	450	300	350	305
		Min. assembly length	450	75	50	205
		Quality score threshold	15	19	0	15
		p-value	0.01	0.01	0.01	0.01
Quality filtering	Swarm	Expected no. of errors per read	0.5	0.5	0.25	0.5
		Min. sequence length	75	75	75	75
Primer removal	Cutadapt	No. of mismatches	0.10	0.10	0.10	0.10
OTU clustering	Swarm	Cluster radius	1	1	1	1
Taxonomic annotation	BLASTN	Max e-value	1e-5	1e-5	1e-10	1e-20
		Word size	20	20	11	30
		Min. Percent ID	97	80	80	97
		Database	GG	nt	nt	nt
		Max target sequences	100	100	100	500
Lowest common ancestor	MEGAN	Max e-value	1e-25	1e-25	1e-25	1e-25
		Min. bitscore	160	100	100	140
		LCA percent	85	85	85	70

GG=GreenGenes, nt= nucleotide BLAST database

Table 2: Overview of taxonomic grouping at each level of the data post occupancy modeling and quality check but pre filtering by Kendall's Tau cut-off limit, see Supplementary Information Fig. 3).

Taxonomy	16S	18S	COI	12S	Sum
**Order**	47	283	159	20	509
**Family**	58	443	269	24	794
**Genera**	93	701	379	30	1,203
**Species**	109	997	491	32	1,629
**# of OTU**	3945	6600	7899	167	18,611

Table 3: Top 10 taxa for all subnetworks represented in Fig. 1a ranked by their connectivity measure.

Taxa	Subnetwork	Trophic level	Connectivity index	Rank within network
**Balaenopteridae**	Blue	4	16.53	1
**Urechidae**	Blue	3	15.97	2
**Chrysolepidomonadaceae**	Blue	1	15.96	3
**Pelagophyceae**	Blue	1	15.84	4
**Rhopalodiaceae**	Blue	1	15.83	5
**Sugiuridae**	Blue	3	15.83	6
**Opheliidae**	Blue	3	15.75	7
**Planctomycetales**	Blue	0	15.39	8
**Rathkeidae**	Blue	3	15.23	9
**Gonyaulacaceae**	Blue	2	14.94	10
**Rhodospirillaceae**	Grey	2	34.37	1
**Polycystinea**	Grey	3	34.33	2
**Carangidae**	Grey	3	34.32	3
**Otariidae**	Grey	4	34.24	4
**Goniodomataceae**	Grey	2	34.23	5
**Metridinidae**	Grey	3	34.21	6
**Crypthecodiniaceae**	Grey	2	33.87	7
**Ellobiopsidae**	Grey	3	33.74	8
**Paraphysomonadaceae**	Grey	1	33.38	9
**Deltaproteobacteria**	Grey	0	33.33	10
**Rhizosoleniaceae**	Yellow	1	16.48	1
**Diplopsaliaceae**	Yellow	2	16.41	2
**Ploima**	Yellow	3	16.31	3
**Cryptosporidiidae**	Yellow	3	15.94	4
**Ellisellidae**	Yellow	3	15.92	5
**Echiuridae**	Yellow	3	15.91	6
**Clausidiidae**	Yellow	3	15.91	7
**Hemiaulaceae**	Yellow	1	14.99	8
**Oxytrichidae**	Yellow	3	14.44	9
**Bellerocheaceae**	Yellow	1	14.36	10
**Marynidae**	Orange	3	19.60	1
**Pandeidae**	Orange	3	19.52	2
**Rhynchomolgidae**	Orange	3	19.49	3
**Vannellidae**	Orange	3	19.46	4
**Echinoidea**	Orange	3	19.37	5
**Didiniidae**	Orange	3	19.30	6
**Hemidiscaceae**	Orange	1	19.14	7
**Merlucciidae**	Orange	3	18.13	8

**Cordycipitaceae**	Orange	0	17.77	9
**Thigmophryidae**	Orange	3	17.55	10
**Doliolidae**	Green	3	18.38	1
**Eucalanidae**	Green	3	18.36	2
**Pholoidae**	Green	3	18.35	3
**Oncaeidae**	Green	3	18.34	4
**Sabellariidae**	Green	3	18.20	5
**Basidiobolaceae**	Green	0	18.07	6
**Biddulphiaceae**	Green	1	17.90	7
**Syllidae**	Green	3	17.79	8
**Heteronemertea**	Green	3	17.75	9
**Spionidae**	Green	3	17.53	10
**Sessilia**	Black	3	7.45	1
**Chromulinales**	Black	1	7.42	2
**Gymnodiniaceae**	Black	2	7.37	3
**Scytosiphonaceae**	Black	1	7.28	4
**Peronosporales**	Black	0	7.26	5
**Balanidae**	Black	3	7.23	6
**Laminariaceae**	Black	1	7.19	7
**Saprolegniaceae**	Black	0	5.95	8
**Callyspongiidae**	Black	3	5.08	9
**Cephalothricidae**	Black	3	4.06	10
