# Supplementary material for: Environmental DNA reveals seasonal shifts and potential interactions in a marine community
Source: Nat Commun. 2020 Jan 14;11:254. doi: 10.1038/s41467-019-14105-1 (PMC6959347; doi:10.1038/s41467-019-14105-1)
Supplement: Supplementary file 3 — Reporting Summary [file 41467_2019_14105_MOESM3_ESM.pdf]

nature research

Corresponding author(s): Anni Djurhuus, Collin Closek, Mya Breitbart

Last updated by author(s): Mar 1, 2019

Reporting Summary

Nature Research wishes to improve the reproducibility of the work that we publish. This form provides structure for consistency and transparency in reporting. For further information on Nature Research policies, see Authors & Referees and the Editorial Policy Checklist.

Statistics

For all statistical analyses, confirm that the following items are present in the figure legend, table legend, main text, or Methods section.

n/a	Confirmed	
☐	☒	The exact sample size (*n*) for each experimental group/condition, given as a discrete number and unit of measurement
☐	☒	A statement on whether measurements were taken from distinct samples or whether the same sample was measured repeatedly
☐	☒	The statistical test(s) used AND whether they are one- or two-sided<br>*Only common tests should be described solely by name; describe more complex techniques in the Methods section.*
☐	☒	A description of all covariates tested
☐	☒	A description of any assumptions or corrections, such as tests of normality and adjustment for multiple comparisons
☐	☒	A full description of the statistical parameters including central tendency (e.g. means) or other basic estimates (e.g. regression coefficient) AND variation (e.g. standard deviation) or associated estimates of uncertainty (e.g. confidence intervals)
☐	☒	For null hypothesis testing, the test statistic (e.g. *F*, *t*, *r*) with confidence intervals, effect sizes, degrees of freedom and *P* value noted<br>*Give P values as exact values whenever suitable.*
☒	☐	For Bayesian analysis, information on the choice of priors and Markov chain Monte Carlo settings
☐	☒	For hierarchical and complex designs, identification of the appropriate level for tests and full reporting of outcomes
☒	☐	Estimates of effect sizes (e.g. Cohen's *d*, Pearson's *r*), indicating how they were calculated

Our web collection on statistics for biologists contains articles on many of the points above.

Software and code

Policy information about availability of computer code

Data collection	Resulting sequences were processed through the banzai pipeline, a Unix shell script. In combination with the pipeline we used PEAR, Swarm, and VSEARCH v1.8.0 packages to assemble, cluster, and filter paired-end sequence reads. Taxonomic annotations were performed with GreenGenes 13.5 or GenBank nr BLASTN databases. Annotations were interpreted through MEGAN6. We used a site-occupancy model to filter low probability occurrences from the dataset, removed fraction of each OTU abundance contributable to contamination, and removed samples that had high dissimilarity between sister replicates. Created a taxon-specific abundance index scaled 0-1 for each taxon. A mean ensemble index was calculated for any taxa that were identified by more than one locus. Kendall's tau was used as our measure of correlation among taxa and over the eight time points. Decontamination and data processing scripts can be found at: https://github.com/marinebon/eDNA_microbes_whales
Data analysis	Weighted correlation networks were calculated using all pairwise Kendall's tau correlations between all taxa across all samples. Taxa were organized into networks using topological overlap measure. To associate taxa to environmental variables a sparse partial least squares (sPLS) analysis was conducted with the mixOmics R package. R code for network and sPLS analyses are located in: https://github.com/marinebon/eDNA_microbes_whales

For manuscripts utilizing custom algorithms or software that are central to the research but not yet described in published literature, software must be made available to editors/reviewers. We strongly encourage code deposition in a community repository (e.g. GitHub). See the Nature Research guidelines for submitting code & software for further information.

Data

Policy information about availability of data

All manuscripts must include a data availability statement. This statement should provide the following information, where applicable:

- Accession codes, unique identifiers, or web links for publicly available datasets
- A list of figures that have associated raw data
- A description of any restrictions on data availability

The data have been deposited with links to BioProject accession number PRJNA433203 in the NCBI BioProject database (https://www.ncbi.nlm.nih.gov/bioproject/).

Field-specific reporting

Please select the one below that is the best fit for your research. If you are not sure, read the appropriate sections before making your selection.

☐ Life sciences ☐ Behavioural & social sciences ☒ Ecological, evolutionary & environmental sciences

For a reference copy of the document with all sections, see nature.com/documents/nr-reporting-summary-flat.pdf

Ecological, evolutionary & environmental sciences study design

All studies must disclose on these points even when the disclosure is negative.

Study description	In this manuscript we amplify four gene regions from eDNA to investigate cross-trophic level biodiversity fluctuations and the responses of diverse taxa to environmental changes. Our time-series sampling encompassed an 18-month period in Monterey Bay, California, and we used correlations among eDNA abundance indices of hundreds of taxa detected from all domains of life to infer dynamic interaction webs.
Research sample	Seawater samples were collected approximately bimonthly for 18 months (n = 8 time points, April 2015 – December 2016) from a long-term monitoring station in Monterey Bay, California, USA. These 1-liter seawater samples were filtered onto a 0.22 µm polyvinylidene difluoride (PVDF) membrane filter to concentrate environmental DNA (eDNA). Four conserved gene regions were selectively amplified. In total, 96 environmental samples were processed: 8 time points x 3 replicates x 4 loci.
Sampling strategy	Sampling was carried out on the R/V Rachel Carson and Western Flyer bimonthly at the permanent Monterey Bay (MB) time series station, C1 (36.797°N, 121.847°W). Samples were collected via Niskin bottles on a rosette at the surface (0-1 m depth) of the water column. Samples were chosen to represent differing environmental “seasonal” conditions throughout the 18-month time-series to profile the changes in associated community assemblages.
Data collection	Multilocus amplicon sequencing of four genetic loci (16S ribosomal RNA (rRNA), 18S rRNA, cytochrome c oxidase I (COI), and 12S rRNA) was conducted with the Illumina MiSeq sequencing platform. Environmental variables (including water column temperature, salinity, dissolved oxygen, chlorophyll a, and nitrate) were measured in situ or from seawater samples at all sampling time points via a Seabird conductivity, temperature, depth (CTD) instrument.
Timing and spatial scale	Samples were collected at 8 time points, April 2015 – December 2016, approximately bimonthly for 18 months. Time points were chosen as representatives of the seasonal oceanographic conditions.
Data exclusions	Replicates and taxa that did not meet robust filtering standards were removed from subsequent analyses.
Reproducibility	Replicate and control samples were processed to ensure data quality as well as reproducibility of results. In addition, all processing scripts for analyzing the data have been made publicly available to enable reproducibility.
Randomization	During the processing of samples as well as library preparation, samples were randomized, and tags were randomly assigned.
Blinding	We did not use blinding in our study, as it was cost prohibitive to do so considering the size of the study and costs to conduct the study. Although, we recognize that having another group perform our study would have provided greater confidence. We did however have three groups work on the different loci with the same samples to ensure consistency amongst methods. We also randomized the samples to reduce bias. Together this created a pseudo-blind test, as the resulting data from each group was brought together in the end for the subsequent analyses.

Did the study involve field work? ☒ Yes ☐ No

Field work, collection and transport

Field conditions	Field conditions varied during 8 time points, but all filter collections took place within the wet lab space and all filtering materials were sterilized prior to collection.
Location	Monterey Bay (MB) time series station, C1 (36.797°N, 121.847°W). Samples were collected via Niskin bottles on a rosette at the surface (0-1 m depth) of the water column.

Access and import/export	No permits were required to collect the eDNA samples. All samples were seawater and required no additional permissions for access, import, or export.
Disturbance	To the best of our knowledge no disturbance was caused by our study.

Reporting for specific materials, systems and methods

We require information from authors about some types of materials, experimental systems and methods used in many studies. Here, indicate whether each material, system or method listed is relevant to your study. If you are not sure if a list item applies to your research, read the appropriate section before selecting a response.

Materials & experimental systems

n/a	Involved in the study
☒	☐ Antibodies
☒	☐ Eukaryotic cell lines
☒	☐ Palaeontology
☒	☐ Animals and other organisms
☒	☐ Human research participants
☒	☐ Clinical data

Methods

n/a	Involved in the study
☒	☐ ChIP-seq
☒	☐ Flow cytometry
☒	☐ MRI-based neuroimaging
